# Revolutionizing nephrology research: expanding horizons with kidney-on-a-chip and beyond

**DOI:** 10.3389/fbioe.2024.1373386

**Published:** 2024-03-28

**Authors:** Wei Huang, Yi-Yuan Chen, Fang-Fang He, Chun Zhang

**Affiliations:** Department of Nephrology, Union Hospital, Tongji Medical College, Huazhong University of Science and Technology, Wuhan, China

**Keywords:** organs-on-a-chip, kidney-on-a-chip, human-on-a-chip, microfluidics, bioprinting

## Abstract

Organs-on-a-chip (OoC) is a microengineered three-dimensional cell culture system developed for decades. Utilizing microfluidic technology, OoC cultivates cells on perfusable channels to construct *in vitro* organ models, enabling the simulation of organ-level functions under physiological and pathophysiological conditions. The superior simulation capabilities compared to traditional animal experiments and two-dimensional cell cultures, making OoC a valuable tool for *in vitro* research. Recently, the application of OoC has extended to the field of nephrology, where it replicates various functional units, including glomerulus-on-a-chip, proximal tubule-on-a-chip, distal tubule-on-a-chip, collecting duct-on-a-chip, and even the entire nephron-on-a-chip to precisely emulate the structure and function of nephrons. Moreover, researchers have integrated kidney models into multi-organ systems, establishing human body-on-a-chip platforms. In this review, the diverse functional kidney units-on-a-chip and their versatile applications are outlined, such as drug nephrotoxicity screening, renal development studies, and investigations into the pathophysiological mechanisms of kidney diseases. The inherent advantages and current limitations of these OoC models are also examined. Finally, the synergy of kidney-on-a-chip with other emerging biomedical technologies are explored, such as bioengineered kidney and bioprinting, and a new insight for chip-based renal replacement therapy in the future are prospected.

## 1 Introduction

Organs-on-a-chip (OoC) technology, a microphysiological system (MPS) based on microfluidics, has emerged as a revolutionary approach for culturing living human cells within microchannels to replicate organ-level physiological and pathophysiological functions *in vitro* (Huh et al., 2013; Solanki et al., 2020; Zhang et al., 2020). These intricate microdevices, fabricated through photolithography to create channels on polydimethylsiloxane (PDMS) or various thermoplastics such as polymethyl methacrylate, polycarbonate and cyclic olefin copolymer, house various types of cells within these channels, emulating organ functionality by recreating tissue-tissue interfaces and facilitating communication between two or more tissues (Leung et al., 2022). Due to their capacity for accurate organ simulations, OoCs have found extensive application in studying tissue development, modeling disease processes, and evaluating drug toxicity. Notably, Huh D *et al.* pioneered the development of a lung-on-a-chip to model the alveolar-capillary barrier, inspiring the creation of numerous other OoCs (Huh et al., 2010).

The kidneys, responsible for regulating water, electrolytes, and acid-base balances, carry out these vital functions through millions of nephrons, each comprising the glomerulus and renal tubules (Gueutin et al., 2012). Abnormalities in any component of the nephron can lead to renal dysfunction. Moreover, the kidney plays a crucial role in drug excretion. The accumulation of drugs can exert toxic effects on this vital organ and drug-induced kidney injury (DIKI) accounted for 18%–27% of the etiology of acute kidney injury (AKI), making renal evaluation a pivotal aspect of drug development (Taber and Pasko, 2008; Radi, 2019). The previous gold standard for studying kidney diseases and drug nephrotoxicity was animal-based *in vivo* experiments, which were limited to time-consuming, high cost, and species differences (Knight, 2008). The data indicate that 10% of failure during both preclinical and clinical phases of drug development can be attributed to DIKI (Cook et al., 2015). Traditional two-dimensional (2D) cell culture, lacking the physiological microenvironment, such as extracellular matrix (ECM) and fluid shear stress (FSS), fails to retain the indispensable *in vivo* characteristics of cells, rendering it an inadequate predictive model (Kishi et al., 2021). OoC technology addresses this limitation by providing fluid flow, mechanical signals, and organ-level tissue interface, enabling the recreation of three-dimensional (3D) organ structures and physiological environments while facilitating dynamic observations of physiological or pathophysiological processes (Wu et al., 2020). These advantages position OoCs as advanced *in vitro* models, widely applied in kidney-related studies.

**FIGURE 1:**
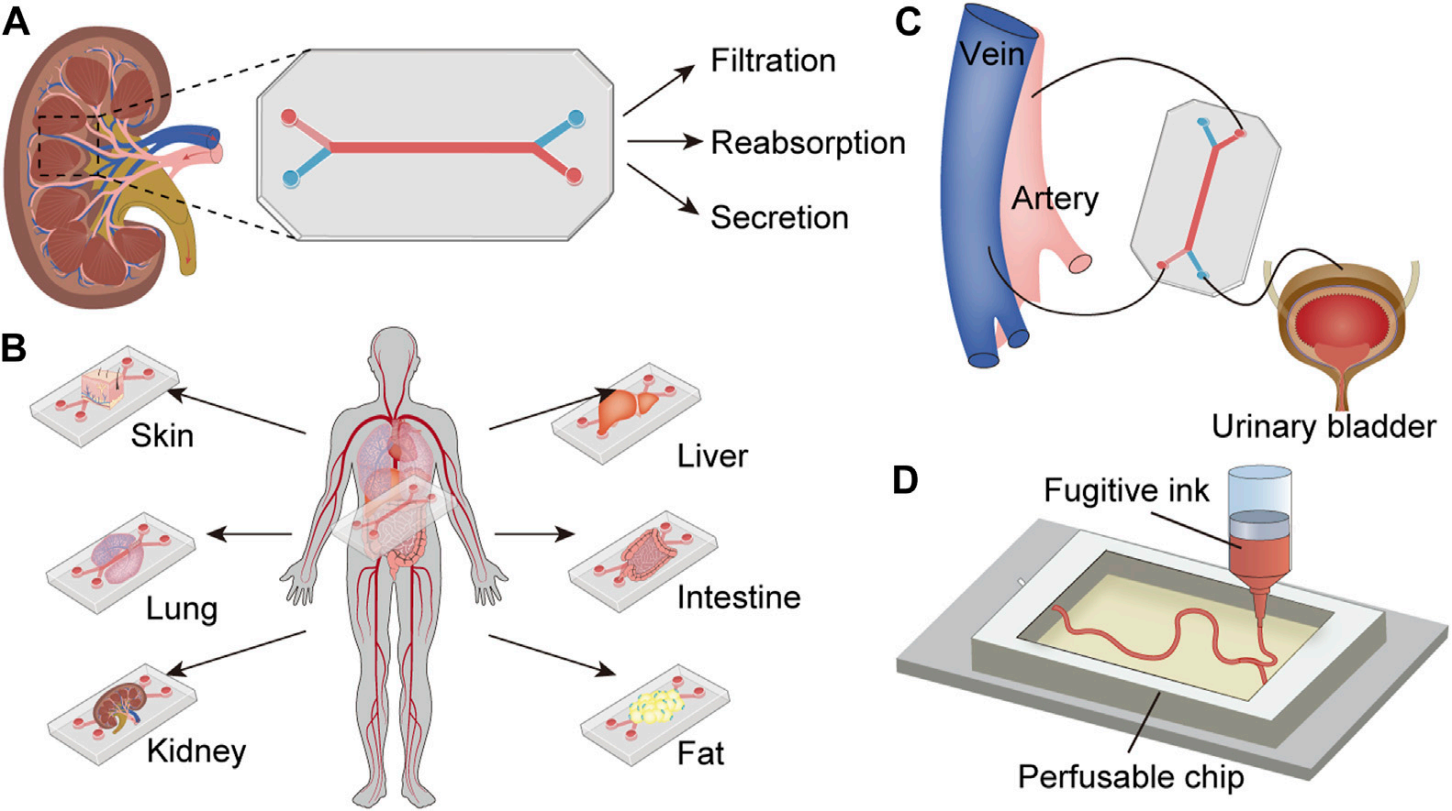
Profile for the content of the review. **(A)** Functional kidney units-on-a-chip (generic model): Kidney cells are seeded in the microchip, which is separated into two channels, each infused with fluid to mimic blood and urine, thereby replicating the structure and function of the kidney unit. **(B)** Multi-organs-on-a-chip including kidney: Several organ-on-a-chips are integrated into one model to simulate the interactions among multiple organs. **(C)** Chip-based bioengineered kidney: The microchip replaces the kidney to remove metabolic waste from the artery and deliver purified blood into the vein, producing urine flows to the urinary bladder. **(D)** Bioprinted kidney-on-a-chip: The kidney-like structure is printed with fugitive ink in the engineered ECM, which is then integrated into the microchip perfused with kidney cells.

Over the past decade, biomimetic microfluidic models that mimic the structure of various functional kidney units, including the glomerulus, proximal tubule, distal tubule, collecting duct, and even entire nephrons, have been developed to construct kidney disease models and assess drug-induced nephrotoxicity (Ashammakhi et al., 2018). Multiplex microfluidic devices have also emerged to investigate interactions between multiple organs, including the kidney (Lee and Sung, 2017). Furthermore, advancements in biomedical technologies, such as bioengineered kidneys and 3D bioprinting, also promote the innovation of OoC technology (Fissell and Roy, 2009; Davoodi et al., 2020). The convergence of these diverse technologies is expected to overcome current limitations and guides the future development of the kidney-on-a-chip models. Here, we describe the characteristics of these chips and their applications in drug testing and kidney disease models (Table 1). Specifically, we focus on the essential aspects of device design, operation, and measurement for each functional unit, emphasizing the importance these factors in accurately replicating renal functions. We also summarize the recent research progress in the field of the kidney-on-a-chip and propose future development prospects. The main content of this review is summarized in Figure 1.

**TABLE 1:** The characteristics of several functional kidney units-on-a-chip models.

Chips	Cell components	Structures	Functions	Applications	Limitations	Solutions
Glomerulus-on-a-chip	GECs and podocytes	GECs and podocytes were implanted on opposite sides of the porous membrane to replicate the three-layer structure of GFB.	The channel housing GECs emulates the vascular lumen, while the channel containing podocytes simulates the urinary lumen. Fluid within the vascular lumen can traverse the basement membrane, mirroring the filtration process of the GFB.	Disease model: diabetic nephropathy (Wang et al., 2017), hypertensive nephropathy (Zhou et al., 2016; Chen et al., 2018; Doi et al., 2023); drug nephrotoxicity test: adriamycin (Musah et al., 2017)	Podocytes are difficult to culture *in vitro*	1) Obtaining mature podocytes from iPSCs
						2) Simulating the special microenvironment around the glomerulus such as FSS to promote podocyte differentiation
						3) Deriving podocytes from amniotic fluid
Proximal tubule-on-a-chip	PTECs	PTECs are cultured on a membrane that divides the microdevice into two channels. The fluid in apical channel mimics urine and the fluid in basolateral channel resembles blood	Substances in blood and urine were exchanged through the porous membrane, simulating the reabsorption and secretion processes of PTECs	Disease model: renal lithiasis (Wei et al., 2012), renal interstitial fibrosis (Zhou et al., 2014), Lowe syndrome (Naik et al., 2021); drug nephrotoxicity test: cisplatin (Jang et al., 2013; Vormann et al., 2021), tenofovir (Vormann et al., 2021), tobramycin (Vormann et al., 2021), cyclosporine A (Vormann et al., 2021), gentamicin (Ioannidis et al., 2022), polymyxin B (Jing et al., 2022), adriamycin (Jing et al., 2022) and sunitinib (Jing et al., 2022); transporter study: P-glycoprotein (Vriend et al., 2018), MRP2/4 (Vriend et al., 2018), OCT2 (Nieskens et al., 2020)	PTECs polarization and drug transporter expression are limited	1) Obtaining polarized PTECs from iPSCs
						2) Developing conditional immortalized PTECs with overexpressions of drug transporters
Nephron-on-a-chip	GECs, podocytes, VECs and PTECs	The nephron-on-a-chip consists of two distinct sections: a glomerular component composed of GECs and podocytes, and a tubular segment comprising VECs and PTECs. These two segments are interconnected but separated into the distinct blood and urinary channel by a porous membrane	As fluid flows through these channels, substances are exchanged between them. The glomerular section performs the function of ultrafiltration, while the renal tubule section handles reabsorption and secretion processes. They replicate the overall functionality of a nephron	Drug nephrotoxicity test: cisplatin (Qu et al., 2018; Zhang and Mahler, 2023),adriamycin (Qu et al., 2018; Zhang and Mahler, 2023)	1) Challenges in vascularization.	Developing kidney organoids to promote regeneration and functionalization of nephrons
					2) Limited regenerative potential of nephrons	
					3) Complexity of replicating renal metabolic and endocrine functions	
Liver-kidney-on-a-chip	Hepatocytes, Kupffer cells, stellate cells, endothelial cells, VECs, PTECs, etc.	Liver and kidney cells were cultivated separately to create liver analogs and kidney analogs within their respective culture chambers. These two chambers were interconnected through microfluidic channels	The liver-kidney-on-a-chip can replicate both liver metabolism and kidney excretion concurrently, making it a valuable tool for studying the collaborative impact of liver and kidney fuctions on drugs	Drug nephrotoxicity test: ifosfamide (Choucha-Snouber et al., 2013; Li et al., 2018), aristolochic acid (Chang et al., 2017), verapamil (Li et al., 2018), Aflatoxin B1 (Theobald et al., 2018), cyclosporine A (Lin et al., 2020)	1) The single cell line cannot fully summarize liver and kidney function	1) Using different cell types to build complete functional units of liver and kidney
					2) The precision of the adjustment of physical and chemical signals in different chambers needs to be improved	2) Introducing the more precisely controlled systems, such as bioprinted OoCs and intelligent passive microfluidic systems

GEC, glomerular endothelial cell; GFB, glomerular filtration barrier; iPSC, induced pluripotent stem cell; FSS, fluid shear stress; VEC, vascular endothelial cell; PTEC, proximal tubule epithelial cell; MRP2/4, multidrug resistance-associated protein 2/4; OCT2, organic cation transporter 2; OoC, organs-on-a-chip.

## 2 Functional kidney units-on-a-chip

### 2.1 Glomerulus-on-a-chip

**FIGURE 2:**
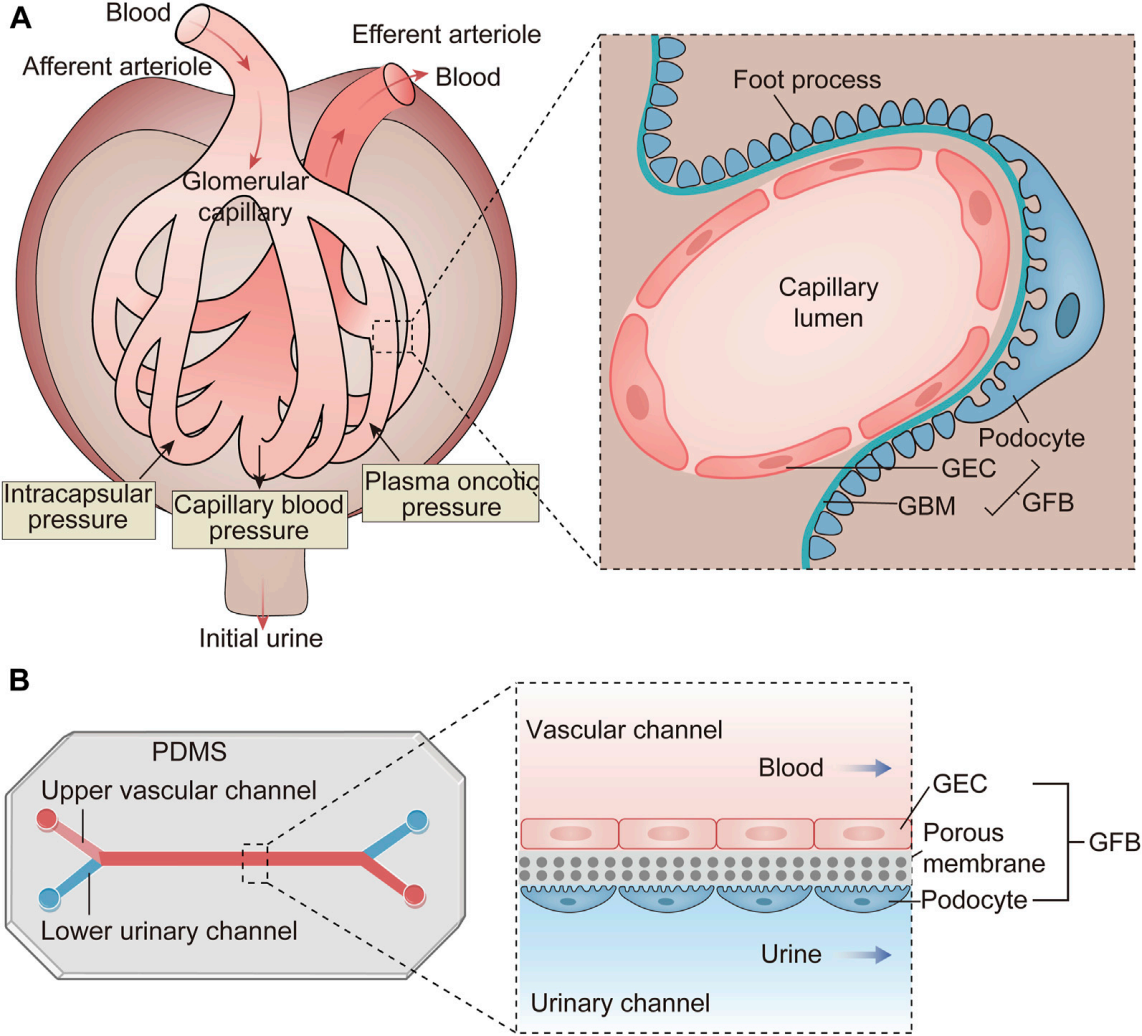
The structure and function of the glomerulus and the schematic diagram of the glomerulus-on-a-chip. **(A)** The glomerulus is a cluster of capillaries featuring an afferent arteriole and an efferent arteriole at either end. Its primary function involves hemofiltration of incoming blood. This filtration occurs under the influence of various pressures, including capillary blood pressure, intracapsular pressure, and plasma oncotic pressure. The resulting filtrate is termed initial urine and is directed into Bowman’s capsule. The structural foundation for this function lies in the GFB, comprising GECs, the GBM and podocytes. **(B)** The glomerulus-on-a-chip is constructed by fabricating microchannels on PDMS. GECs are implanted into the upper vascular channel, while podocytes are placed in the lower urinary channel. A porous membrane separates these two cell types, mimicking the three-layer structure of the GFB. As blood and urine flow through this microdevice, substances are exchanged between the two channels, simulating the filtration process within the glomeruli. GEC, glomerular endothelial cell; GBM, glomerular basement membrane; GFB, glomerular filtration barrier; PDMS, polydimethylsiloxane.

A common physiopathologic feature of chronic kidney diseases, regardless of their underlying causes, is the loss of functional glomeruli. The glomerulus, a compact cluster of capillaries, plays a critical role in filtering fluid and electrolytes from the blood while retaining plasma proteins. This unique hemofiltration function relies on the integrity of glomerular filtration barrier (GFB), composed of glycosylated glomerular endothelial cells (GECs), highly differentiated podocytes, and the glomerular basement membrane (GBM) situated between them (Figure 2A). Disruption of the GFB integrity leads to proteinuria, a significant factor accelerating nephropathy progression. Therefore, the development of effective GFB models is essential for studying glomerular diseases.

Glomerulus-on-a-chip models can be constructed using intact glomerular tissue within microfluidic channels. More commonly, these models consist of GECs and podocytes cultured within microfluidic channels, separated by a porous, flexible membrane that simulates hemofiltration as fluid flows through it (Figure 2B) (Zhou et al., 2016). For the interactions between GECs and podocytes are crucial for functional GFB, the synthetic membranes employed for glomerulus-on-a-chip must possess pores conductive to the free exchange of media and growth factors to achieve the intercellular crosstalk. The main challenge of this model is culturing podocytes *in vitro*. Podocytes, highly differentiated cells covering the GBM surface, play a vital role in GFB by regulating its selective permeability (May et al., 2014). Due to species-specific differences, it is preferable to evaluate human primary cultured podocytes rather than relying on animal-derived cell lines or conditionally immortalized podocytes (Li et al., 2016). However, primary podocytes often undergo de-differentiation after isolation from human kidneys, making it challenging to obtain mature, differentiated podocytes. Musah S *et al*. successfully addressed this challenge by differentiating human induced pluripotent stem cells (iPSCs) into mature podocytes on microfluidic chips (Musah et al., 2017). They simulated the *in vivo* environment of podocyte differentiation more accurately by adding ECM components. The unique environment around the glomerulus, such as FSS, also has an important effect on podocyte differentiation. One study demonstrated that FSS combined with retinoic acid could induce the differentiation of primary podocytes within microfluidic devices (Yang et al., 2017). In addition, it has been reported that amniotic fluid-derived podocytes, which can be expanded for multiple generations before differentiation, offer a more convenient alternative to immortalized podocytes (Da Sacco et al., 2013). There are still some challenges in the formation of podocyte monolayer *in vitro*. Porous membranes are typically coated with ECM such as collagen I or IV to promote podocyte adhesion. Studies have shown that utilizing different ECM compositions, particularly laminin 521, enhanced podocyte attachment and proliferation (Doi et al., 2023). Optimal filtration flow rates also play a crucial role in facilitating podocyte monolayer formation. In addition, intercellular crosstalk, particularly VEGF-induced GECs maturation, significantly impacts podocyte adhesion; inadequate VEGF treatment may result in podocyte detachment from the membrane (Li et al., 2016).

Glomerulus-on-a-chip has been applied to construct several disease models. Wang L *et al.* cultured isolated intact glomerular microtissues on a microfluidic device and exposed them to high glucose medium at concentration of 5 mM and 30 mM, thus establishing an *in vitro* model of diabetic nephropathy (DN) (Wang et al., 2017). Their results indicated that this DN microdevice replicated high glucose-induced GFB dysfunction, including increased albumin permeability, reduced zonula occludens-1 (ZO-1) expression, excessive reactive oxygen species production, and increased podocyte motility. This approach provides a novel strategy for elucidating pathological mechanisms of DN. Chen TH *et al.* fabricated a microfluidic platform to investigate the mechanisms of hypertension-induced glomerular leakage (Chen et al., 2018). The porous membrane was positioned between two acrylic chambers, and the culture medium was delivered using peristaltic pumps. Transmembrane pressure between the chambers was regulated by the peristaltic pumps and monitored through pressure sensors. They found high transmembrane pressure exceeding 30 mmHg damaged podocyte filtration function by reducing synaptopodin expression and disrupting the actin cytoskeleton. Zhou M *et al.* mimicked hypertensive nephropathy by adjusting the perfused culture medium’s flow rate in a glomerulus-on-a-chip (Zhou et al., 2016). The baseline perfusion rate was set at 5 μL/min, with hyperperfusion defined as 10 and 15 μL/min. They found that under hyperperfusion conditions, the excretion of small molecular-weight proteins and albumin increased. In endothelial cells, the cytoskeleton of F-actin rearranged, the expressions of F-actin and CD31 decreased, and the injurious marker von Willebrand factor increased. In podocytes, F-actin and actin-associated protein synaptopodin decreased, and the expressions of slit diaphragm proteins nephrin and podocin also decreased. Doi K *et al.* developed a glomerulus-on-a-chip that precisely regulated fluid flow and filtration pressure by controlling air pressure to explore the effects of filtration stimulation on podocyte differentiation (Doi et al., 2023). Their results showed that filtration stimuli with a pressure of 0.10 kPa induced and maintained the foot processes of podocytes by upregulating the expression of synaptopodin, CD2-related proteins, and vascular endothelial growth factor-A, and could affect the response of podocytes to drugs. This model provided new insights into the mechanobiology of glomerular cells.

Gomerulus-on-a-chip models are also valuable tools for drug toxicity testing. Musah S *et al.* established a microfluidic device with iPSC-derived podocytes and primary human GECs (Musah et al., 2017). This MPS filtrated inulin from the vascular compartment into the urinary channel while retaining albumin in the vascular channel, recapitulating the selective permeability of the human glomerulus. When exposed to adriamycin, podocytes detached into the urinary channel, and albumin was excreted from the vascular channel into the urinary channel. This glomerulus-on-a-chip replicated adriamycin-induced podocyte injury and drug toxicity-induced albuminuria. Petrosyan A *et al.* explored co-culturing podocytes and GECs in a microfluidic chip without using any artificial membrane. Their results showed that these two cells maintained their phenotype over an extended period, and they exhibited the remarkable capability to produce essential ECM components such as collagen IV trimers and laminin. This approach led to the formation of an ECM layer, effectively addressing a long-standing limitation of prior glomerulus-on-a-chip models, which lacked a realistic GBM (Petrosyan et al., 2019). This innovative model responded to various injuries, including puromycin aminonucleoside, high glucose, and nephrotoxic serum from patients with membranous nephropathy, demonstrating its potential for clarifying molecular mechanisms of glomerular diseases and testing nephrotoxicity of new drugs.

The destruction of the GFB, particularly the loss of podocytes, is a common pathological feature in many primary and secondary kidney diseases. However, due to the complex and not fully understood pathogenesis, there is currently no specific treatment for glomerular injury in clinical practice. Therefore, accurately simulating the glomerulus and its surrounding microenvironment *in vitro* is a critical focus in nephrology research. Although glomerulus-on-a-chip models have been developed, they still have limitations in replicating certain aspects of the glomerular microenvironment. For instance, the fluid flow rate and filtration pressure in most current models are lower than physiological levels observed *in vivo*. With advancements in engineering approaches, ongoing improvements in glomerulus-on-a-chip technology hold promise for enhancing our understanding of the pathogenesis of various glomerular diseases and providing a platform for exploring novel therapeutic strategies.

### 2.2 Proximal tubule-on-a-chip

The proximal renal tubule plays a pivotal role in drug clearance and reabsorption, making it a primary target for drug-induced nephrotoxicity. The inability of conventional 2D planar cell culture and animal models to accurately predict the effects of drugs on the kidney is the major cause of drug development failure (Tiong et al., 2014). Thus, there is a great need for *in vitro* models of proximal tubules capable of precisely recapitulating human responses in preclinical drug trials.

The proximal tubule handles filtrate flow from GFB, generating FSS on proximal tubule epithelial cells (PTECs). FSS has been proven to be of great physiological significance for PTECs, including actin cytoskeleton, tight junction and adhesive junction formation, regulation of sodium transport, and enhanced albumin absorption (Duan et al., 2008; Duan et al., 2010). Conventional 2D cell cultures fail to replicate this physiological condition, resulting in the loss of PTEC differentiation phenotype and decreased transporter expression, limiting their ability to predict nephrotoxicity. In contrast, OoC technology can recreate FSS within its microfluidic systems, and when supplemented with ECM components, it can simulate a more realistic internal environment. Studies have shown that PTECs cultured in microfluidic devices exhibit upregulated expression of ZO-1, increased numbers of ciliated cells, and enhanced albumin uptake and degradation (Ferrell et al., 2012; Jang et al., 2013).

The apical membranes of PTECs are situated within the proximal tubule lumen, where they are exposed to FSS resulting from filtrate flow. Conversely, the basolateral membranes of PTECs face the renal interstitium and express various transporters. In conventional proximal tubule-on-a-chip models, PTECs are cultured on a membrane that divides the microdevice into two channels. The upper channel exposes the apical membranes of the PTECs to fluid mimicking urine, generating FSS. Meanwhile, the lower channel provides an interstitial region where the basolateral membrane of PTECs interfaces with an internal fluid resembling blood for stimulation and exchange. Moreover, the OrganoPlate represents a novel microfluidic culture system based on the 384-well microtitration plate format, comprising 40 or 96 chips designed for the long-term culture of living cells. This platform features PhaseGuide technology, enabling the layering of the ECM in microchannels without artificial membranes. FSS in the OrganoPlate system can be achieved through gravity-induced fluid flow. It facilitates the co-culture of renal tubules and vessels under FSS and has been utilized in several proximal tubule-on-a-chip studies (Vriend et al., 2018; Naik et al., 2021; Vormann et al., 2021). Current proximal tubule-on-a-chip models face challenges particularly in achieving epithelial cell polarization and transporter expression. Human primary PTECs are often considered the gold standard for *in vitro* modeling of proximal tubules. Still, they exhibit significant variability depending on individual differences and have limited self-renewal capacity, leading to lower model reproducibility (Sakolish et al., 2018). Immortalized PTECs, another commonly used cell line, lack apical-basolateral polarization and the expression of several drug-associated transporters, such as the solute carrier family 22 transporter family (Jenkinson et al., 2012). One study derived PTECs from kidney organoids differentiated from human iPSCs and found that they showed significant transporter upregulation and improved drug uptake capacity compared to immortalized PTECs (Aceves et al., 2022). Additionally, a microfluidic device based on conditionally immortalized PTECs overexpressing organic anion transporter 1 has been developed, displaying normal cell polarization and broad expressions of drug transporters, making it a valuable tool for studying nephrotoxicity and drug-transporter interactions (Vriend et al., 2018). For more accurate nephrotoxicity predictions, utilizing patients-derived regenerative cells as materials for proximal tubule-on-a-chip holds promise, although experimental exploration of such applications is still pending.

Compared to glomerulus-on-a-chip models, proximal tubule-on-a-chip technology is more mature and has found extensive application in renal drug transport and nephrotoxicity assessment. Jang KJ *et al.* conducted the first toxicity study using primary PTECs in a microfluidic device (Jang et al., 2013). The researchers employed a porous membrane seeded with primary PTECs to partition the microdevice into an apical channel and a basolateral channel, mirroring the *in vivo* structure of the proximal tubule. FSS was applied to the apical channel, while solutes and drugs were introduced into the basolateral channel to study epithelial transport. This setup allowed for the establishment of cell polarity, as evidence by the cellular normal columnar form and strict localization of Na/K-ATPase on the basolateral surface. Under flow conditions, PTECs exhibited significant functional enhancements, including a 2- to 3-fold increase in Na/K-ATPase and aquaporin 1 (AQP1) expression, over a 2-fold increase in albumin uptake, a 3.5-fold increase in glucose transport, and a 4-fold increase in alkaline phosphatase activity, confirming their physiological functions. Moreover, toxicity assessments revealed a reduction in cisplatin-induced nephrotoxicity and an augmented protective effect of cimetidine against cisplatin-induced cytotoxicity compared to static conditions. In another study, primary PTECs were cultured into a normal monolayer form and then seeded into an MPS, forming a 3D tubular structure under fluid conditions (Weber et al., 2016). Expression of CD13 and E-cadherin demonstrated cell self-assembly, while AQP1 expression confirmed proximal tubule origin. Apical localization of ZO-1 and basolateral localization of Na+/K+ ATPase further validated cell polarization, highlighting the physiological relevance of PTECs. Various physiological functions of the proximal tubules in this MPS were assessed by perfusing culture medium containing different substances, including glutathione reclamation, glucose reabsorption, vitamin D bioactivation, and secretion of para-aminohippurate and indoxyl sulfate. This study is the first report that models the transport, reabsorptive, and metabolic processes of PTECs *in vitro* and may provide a valuable platform for screening new drugs.

The secretory process of the renal tubules has also been modeled in OoCs. Jansen J *et al.* demonstrated that PTECs, seeded in bioengineered hollow fibers perfused with flow, actively secreted protein-bound uremic toxins through specific apical and basolateral transporters (Jansen et al., 2016). Vriend J *et al.* developed a microfluidic proximal tubule model using a dual-channel OrganoPlate to investigate the functions of the efflux transporters P-glycoprotein (P-gp) and multidrug resistance-associated protein 2/4 (MRP2/4) (Vriend et al., 2018). The OrganoPlate comprised a gel channel and an apical channel. ECM was introduced into the gel channel, while PTECs overexpressing organic anion transporter 1 were seeded onto the apical channel and gravitationally attached to the ECM. The two channels formed membraneless compartments facilitated by PhaseGuide technology. Under FSS, induced by the passive leveling of culture medium in the apical channel inlets and outlets, PTECs formed a monolayer. Cell polarization was evidenced by the apical localization of F-actin and ZO-1. The expression of P-gp and MRP1/4 in PTECs mirrored that observed in 2D culture. The activity of these two transporters was assessed through fluorescence-based efflux assays of their substrates after incubating PTECs in the presence of various inhibitors. Confocal imaging confirmed that inhibiting P-gp increased calcein retention, while inhibiting MRP2/4 led to glutathione accumulation, validating this model can be applied for screening and detecting drug-transporter interactions. Nieskens TTG *et al.* developed dual-channel proximal tubule-on-a-chips demonstrating the exclusive sensitivity of organic cation transporter 2 (OCT2) on the proximal tubule basolateral membrane to the nephrotoxic drug cisplatin (Nieskens et al., 2020). Each chip comprised two parallel channels coated with collagen IV, situated within a large permeable collagen I matrix. Primary PTECs were seeded into the apical channels, where they adhered to the collagen IV under perfusion of culture medium, forming a tubular monolayer. The basolateral channels were solely perfused with culture medium to simulate the renal interstitium. ZO-1, lotus lectin and primary cilia were localized to the apical membrane, while OCT2 was exclusively expressed in the basolateral membrane. Cell numbers decreased, and barrier integrity was disrupted when cisplatin was injected at a concentration of 25 μM from the basal membrane for 72 h, and this effect could be reversed by the OCT2 inhibitor cimetidine. No toxicity was observed when cisplatin passed through the apical membrane, this was consistent with polarization localization of OCT2. This model illustrated the importance of considering the localization of cationic drug uptake transporters for a more realistic simulation of drug nephrotoxicity. In a high-throughput proximal tubule model established with a OrganoPlate containing 40 chips, four model drugs-cisplatin (5 and 30 mM), tenofovir (15.6 and 1000 mM), tobramycin (7.5 and 15 mM), and cyclosporine A (5 and 30 mM) -were assessed for nephrotoxicity, and the results were consistent with existing animal experimental data. Cell viability, lactate dehydrogenase release, and miRNA expression served as predictors (Vormann et al., 2021). Ioannidis K *et al.* developed a proximal tubule-on-a-chip integrated with microsensors to monitor the real-time metabolic kinetics of gentamicin (Ioannidis et al., 2022). The researchers seeded microvascular endothelial cells and primary PTECs onto the chip to create vascularized kidney organoids, and they simulated physiological perfusion using computational fluid dynamics. To monitor the dynamic metabolism of gentamicin, microsensors were implanted into the renal tubules. The DynamiX platform was then employed to measure levels of oxygen, glucose, lactic acid, and glutamine under continuous perfusion conditions. Additionally, metabolic flux and ATP production within the chip were quantified simultaneously. The researchers identified the critical gentamicin concentration at which proximal tubule polarity was disrupted, which was 20 times lower than its TC50. This study revealed that gentamicin mediated tubule damage by a 3.2-fold increase in glucose uptake, reversing the circulation flux of the tricarboxylic acid cycle, leading a 40-fold enhance in lipid accumulation, and demonstrated the SGLT2 inhibitor empagliflozin inhibited gentamicin toxicity by 10-fold. Jing B *et al* designed a proximal tubule model using an integrated biomimetic array chip to compare the characteristics of several chemotherapeutic drugs (Jing et al., 2022). The chip comprised 24 functional units, each consisting of five layers. The device was positioned on a shaker to regulate the flow rate and FSS by adjusting the shaking angle and frequency. Following 1 week of dynamic culture, primary PTECs exhibited enhanced cell polarization, barrier function and transporter activity compared to HK-2 cells. The toxicity detection showed that polymyxin B induced nephrotoxicity from both the apical and basolateral membranes of PTECs. In contrast, adriamycin and sunitinib induced nephrotoxicity only from the apical membranes of PTECs.

PTECs are crucial targets in various kidney diseases, and proximal tubule-on-a-chip models have proven invaluable in simulating these disease processes. Wei Z *et al.* cultured PTECs in a tubular microfluidic channel forming a polarized monolayer resembling the lumen structure of renal tubules and distribution of Na/K-ATPase demonstrated the functionalization of the tubules. They simulated stone formation by depositing calcium phosphate within the lumen and monitored this process in real-time under a microscope, providing a novel approach for studying the mechanism of renal lithiasis (Wei et al., 2012). Zhou M *et al.* established two microfluidic chips to investigate the role of complement C3a in epithelial-to-mesenchymal transition (EMT) of proximal tubules (Zhou et al., 2014). The researchers designed a 2D microfluidic device consisting of an upper PDMS layer and a lower glass layer. The PDMS layer housed 12 microchannels, each containing three culture chambers seeded with HK-2 cells and a fluid buffer area. Each channel was connected to a syringe pump to control medium flow rate and FSS. The independent channels allowed cells to be incubated under flow conditions with diverse stimuli simultaneously. Successful induction of EMT in HK-2 cells by TGF-β1 demonstrated the usability of the device. When HK-2 cells were exposed to human serum and C3a, apoptosis and a mesenchymal phenotype were observed. Subsequently, they designed a 3D microdevice consisting of three connected units, each comprising a cell culture chamber and five side channels filled with basement membrane extract (BME). Cultured HK-2 cells attached to BME, mimicking the proximal tubule structure *in vivo*. Upon exposure to C3a, the migration of HK-2 cells across BME and transformation into myofibroblasts were visualized in real-time. This study offered a platform for studying renal interstitial fibrosis mechanisms. Sakolish CM *et al.* established a unique microfluidic device for culturing PTECs, which distinguished it from other proximal tubule-on-a-chip models (Sakolish et al., 2019). In addition to the membrane dividing the device into apical and basolateral channels, a small chamber equipped with a polyether thioketone membrane was incorporated into the apical channel to mimic the function of the glomerulus. As the medium flowed through this chamber, macromolecular particles were effectively removed. PTECs were cultured under various fluid flow conditions, including high glucose (30 mM glucose), kidney stones (0.3 mg/mL calcium oxalate), and nephrotoxic drugs (1.4 mM cisplatin and 0.27 mM cyclosporine). The researchers observed stress-related responses, such as α-smooth muscle actin expression, alkaline phosphatase activity, fibrin, and neutrophil gelatinase-associated adiponectin secretion, which played significant roles in these disease conditions. These findings highlighted the involvement of FSS in the pathogenesis of these diseases. Vriend J *et al.* constructed a proximal tubule model using the dual-channel OrganoPlate, which contained 96 chips, and investigated the effects of FSS and primary cilia on PTECs (Vriend et al., 2020). They observed increased albumin uptake, enhanced drug excretion, and changes in PTECs morphology under pulsed FSS resembling physiological conditions. Remarkably, these effects were not affected when primary cilia were knocked out, suggesting that FSS independently induced phenotypic changes in PTECs, not relying on primary ciliary mechanisms. Lowe syndrome, characterized by defects in proximal tubular reabsorption due to mutations in Lowe’s oculocerebrorenal syndrome (OCRL) gene, was successfully modeled by Naik S *et al.* using a proximal tubule-on-a-chip (Naik et al., 2021). The model was built upon a three-lane OrganoPlate containing 40 chips, with PTECs seeded in the top channel, collagen I filling the middle channel, and the medium infused into the bottom channel. The device was positoned on a rocker to induce FSS, facilitating the formation of a tubule structure through gravitational forces. The integrity of the tubule was confirmed using a glucan diffusion assay. PTECs lacking OCRL expression exhibited upregulated transcription factor Snai2 and excessive collagen deposition, shedding light on the interstitial fibrosis mechanism in Lowe syndrome.

### 2.3 Distal tubule-on-a-chip

Efforts have been made to develop distal tubule-on-a-chip models for *in vitro* chronic toxicity testing. Baudoin R *et al.* seeded Madin Darby Canine Kidney (MDCK) cells extracted from canine distal tubules into microfluidic microchambers and evaluated cell growth under 0, 10, 25, and 50 μL/min flow rates (Baudoin et al., 2007). The researchers employed a peristaltic pump to perfuse MDCK cells into a microchip coated with fibronectin to facilitate cell adhesion. These cells were then exposed to a culture medium containing ammonium chloride. The basal metabolism of the MDCK cells, assessed by measuring glucose consumption and ammonia production resulting from glutamine metabolism in the medium, was combined with cell counting to evaluate the viability of the chip. Morphological observations revealed that high flow rates were unsuitable for MDCK cell growth, possibly due to these cells experiencing lower FSS compared to PTECs under physiological conditions. The optimal flow rate appeared to be 10 μL/min and with increasing flow rates, MDCK cells exhibited approximately 1.5 to 2-fold elevated glucose consumption and glutamine production. Moreover, ammonium chloride inhibited MDCK cell proliferation, increased glucose consumption, and decreased ammonia production during perfusion, consistent with previous findings. In addition, this model offered a high surface-to-volume ratio and supported dynamic culture, indicating its potential *in vitro* drug evaluation.

Viral infections often contribute to renal dysfunction, with the distal tubule serving as a crucial target for many viruses. Wang J *et al.* pioneered the construction of a kidney disease model induced by pseudorabies virus (PrV) infection on a microfluidic chip. They dynamically monitored the interaction between the virus and host through a distal tubule-on-a-chip (Wang et al., 2019). The device was partitioned by a porous membrane into a microfluidic channel and a static liquid well, aimed at simulating the fluid environment of the tubular lumen and the interstitium, respectively. MDCK cells, extracted from canine distal renal tubules, grew into monolayers on the porous membrane under FSS, simulating the reabsorption barrier of the distal tubule. The functionalization of these monolayers was confirmed by the expression of ZO-1, microvilli, and acetylated tubulin. The results demonstrated that PrV infection disrupted the reabsorption barrier of the distal renal tubular epithelium, markedly reduced sodium reabsorption capacity (1.53 ± 0.05 mg/chip·14 h to 0.81 ± 0.08 mg/chip·14 h), altered the expression and distribution of sodium transporters in MDCK cells, and increased the sensitivity of cells to angiotensin-converting enzyme 2. These results suggest that impaired serum electrolyte balance due to PrV infection may be attributed, at least in part, to this mechanism. This innovative model paves the way for future research into the pathogenesis of renal dysfunction induced by virus infection.

### 2.4 Collecting duct-on-a-chip

The collecting duct, comprising the cortical collecting duct (CCD) and medullary collecting duct, plays a crucial role in regulating water and electrolyte balance. Luminal microenvironments, including FSS, hormones, and transepithelial osmotic gradient, have been demonstrated to be essential for aquaporin-2 (AQP2) transfer and actin cytoskeleton reorganization in inner medullary collecting duct (IMCD) cells (Jang et al., 2011). Jang KJ *et al.* fabricated a canonical three-layer microfluidic device for IMCD cell culture and investigated the effect of luminal microenvironments (Jang and Suh, 2010). FSS was applied to the apical membrane of the cells using a syringe pump, while adequate air and medium circulation were ensured for the basolateral membrane. The localization of AQP2 in the intracellular vesicle regions and Na/K-ATPase in the basolateral membrane indicated excellent cell polarization. Furthermore, robust cell viability was confirmed by quantifying the number of living/dead cells. Their findings indicated that FSS enhanced cell polarization with appropriate apical and basolateral marker protein localization, increased cell height, rearranged actin cytoskeleton and cell junctional proteins, and induced AQP2 translocation to the apical plasma membrane. Additionally, stimulation with arginine vasopressin and a transepithelial osmotic gradient further promoted AQP2 trafficking to the apical membrane, facilitating water uptake and aldosterone-induced sodium uptake in IMCD cells. Together with these findings, this collecting duct-on-a-chip can imitate various *in vivo* microenvironments, making it valuable for drug screening and exploring the underlying molecular mechanisms regulating water and electrolyte transport.

The principal cells (PCs) in the CCD are responsible for water and Na^+^ reabsorption. In a recent study, immortalized mouse PC-like CCD cells were seeded in a microfluidic channel (Rein et al., 2020). The researchers bioprinted a 3D tubular structure within a silicone gasket on the chip, mimicking a CCD model. Subsequently, the chip was connected to an external cell culture medium to facilitate fluid flow and cell inoculation within the tubule. The formation of epithelial tight junctions and the expression of channel proteins demonstrated the induction of a polarized phenotype in this model. Moreover, the researchers observed the formation of cell doming when perfusion ceased-a structure resulting from water accumulation in the cell monolayer due to Na^+^ and water reabsorption. This doming phenomenon disappeared when Benzamil inhibited Na^+^ absorption, confirming the model’s reabsorption function. The collecting duct-on-a-chip has yet to be used in disease models and drug toxicity detection, requiring further in-depth research in the future.

### 2.5 Nephron-on-a-chip

**FIGURE 3:**
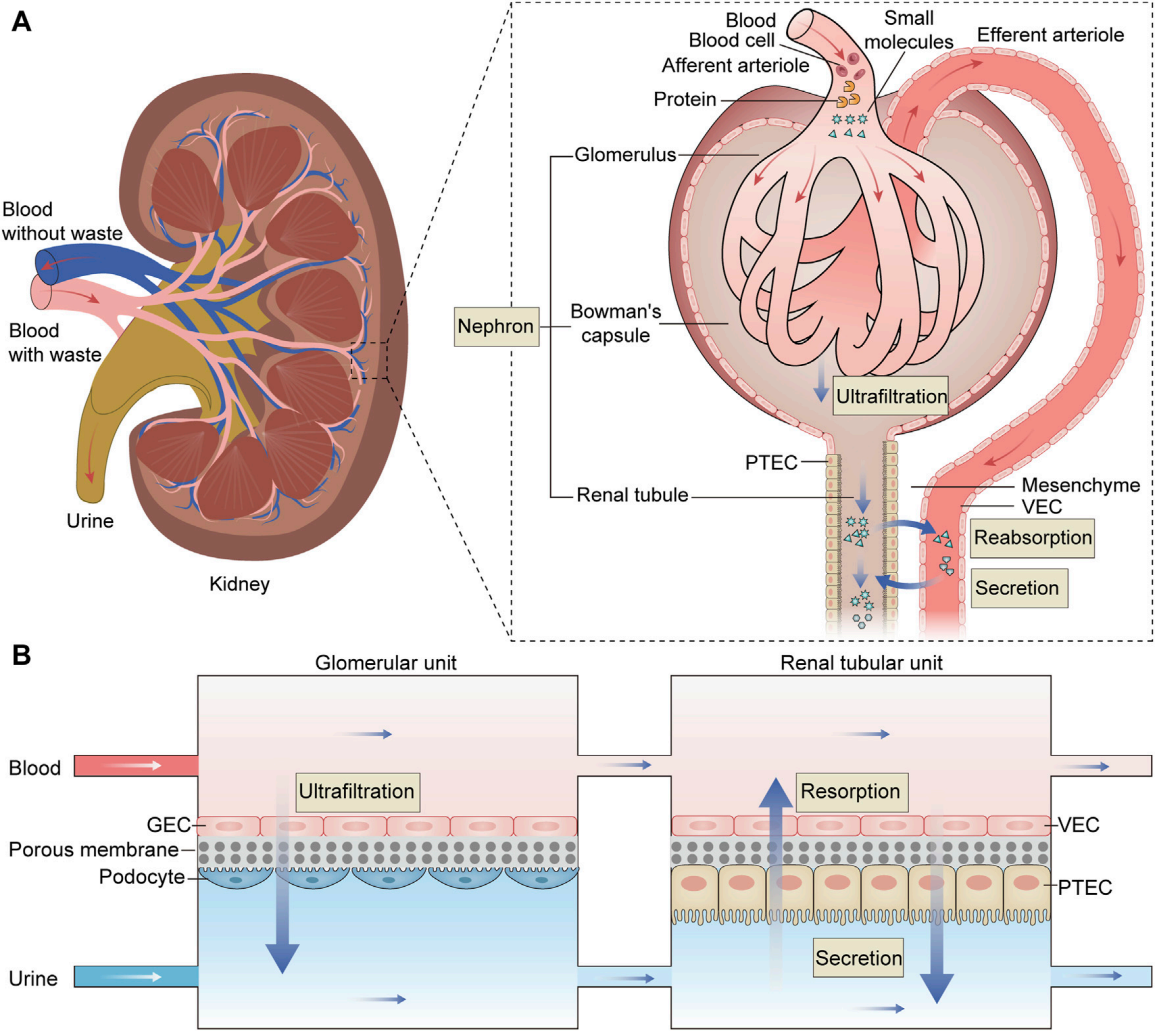
The structure and function of the nephron and the schematic diagram of the nephron-on-a-chip. **(A)** The nephron is the kidney’s fundamental functional unit consisting of glomeruli, Bowman’s capsules, and renal tubules. The glomeruli play the role of ultrafiltration, where they filter the fluid and small molecules from the plasma while retaining essential plasma proteins. The filtered fluid then enters Bowman’s capsule and proceeds into the renal tubule, where it is primarily reabsorbed, particularly in the proximal renal tubules. **(B)** The nephron-on-a-chip is divided into glomerular units and renal tubular units. Within the glomerular unit, GECs and podocytes are placed on opposite sides of a porous membrane. Similarly, in the renal tubular unit,.VECs and PTECs are positioned on opposite sides of a porous membrane. The upper and lower channels within both units are interconnected, allowing for the flow of blood and urine, respectively. These two units work together to simulate the processes of ultrafiltration, reabsorption, and secretion characteristic of a fully functional nephron, acheived through the exchange of substances within the upper and lower channels. PTEC, proximal tubule epithelial cell; VEC, vascular endothelial cell; GEC, glomerular endothelial cell.

Single glomerulus-on-a-chip or renal tubule-on-a-chip models offer partial insights into nephron functions. To comprehensively simulate the structure and function of the kidney, researchers are developing the nephron-on-a-chip, connecting glomerular and renal tubular devices through microchannels to create a complete flow system (Figure 3).

Weinberg E *et al.* initially designed a bioartificial device replicating the three-step system of nephron (Weinberg et al., 2008). This model emulates the entire functional nephron, encompassing filtration, reabsorption, and urine concentration, with flow rates calculated to mimic physiological conditions. While it remains a computational model, it paves the way for the creation of a bioartificial functional nephron. Qu Y *et al.* constructed a nephron-on-a-chip model that encompasses glomerular filtration and renal tubule absorption and secretion (Qu et al., 2018). The nephron-on-a-chip consists of two distinct sections: a glomerular component composed of GECs and podocytes, and a tubular segment comprising VECs and PTECs. These two segments are interconnected but separated into the distinct blood and urinary channel by a porous membrane. Using parallel microchannels to mimic renal blood flow and filtration drainage, this device emulates glomerular size- and charge-selective barrier functions within the “renal corpuscle.” In the “proximal tubule,” the model reabsorbs glucose and secretes para-aminohippuric acid. This nephron model was used to investigate the pathophysiology mechanisms of cisplatin and adriamycin-induced AKI and assess the nephrotoxicity of these drugs. Zhang SY *et al.* integrated glomerulus and proximal tubule microdevices into a multi-layered culture system to construct an *in vitro* nephron model (Zhang and Mahler, 2023). This system consists of human-derived immortalized podocytes, VECs, and tubular cells, successfully simulating serum protein filtration, glucose reabsorption, and creatinine secretion. Researchers employed this model to evaluate drug-induced nephrotoxicity, observing impaired cell attachment, filtration, and reabsorption following exposure to cisplatin and adriamycin. These results underscore the potential of the nephron-on-a-chip as a promising experimental tool.

Vascularization, the drainage of formative urine, and the efficiency of generating new nephrons are the main challenges for the current development of kidney-on-a-chip models. Recent advancements in vascularization and the maturation of glomerular and tubular epithelia within kidney organoids suggest that the development of kidney organoids-on-a-chip may enhance the regenerative capacity of nephrons *in vitro* (Homan et al., 2019). In addition, the reproduction of renal intracellular metabolism and endocrine functions *in vitro* needs to be addressed (Ashammakhi et al., 2018). Simulating the complex renal microenvironment to enable bio-signals and material exchange *in vitro* necessitates careful consideration of interactions between different components of kidney units.

## 3 Multi-organs-on-a-chip including kidney

**FIGURE 4:**
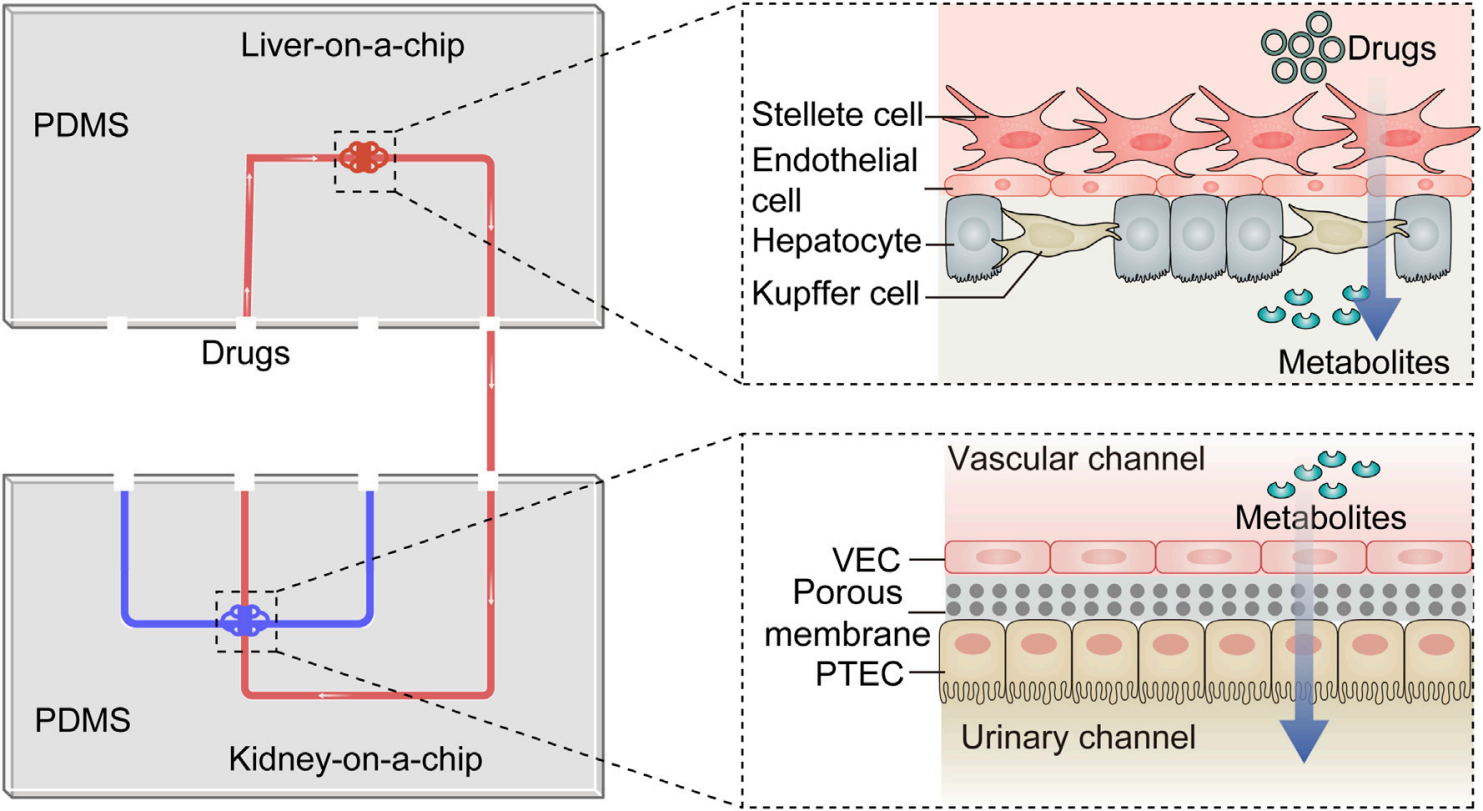
The diagram of the liver-kidney-on-a-chip. The liver-kidney-on-a-chip is composed of two crucial components: the liver-on-a-chip, which incorporates hepatocytes, Kupfer cells, stellate cells and endothelial cells, and the kidney-on-a-chip, housing VECs and PTECs. Together, these components create an integrated platform. Within this system, drugs are undergo metabolism within the liver-on-a-chip, and their resulting metabolites are subsequently excreted into the kidney-on-a-chip. This dynamic setup emulates the *in vivo* drug transformation process and is particularly valuable for investigating the influence of liver metabolism on drug-induced nephrotoxicity. PTEC, proximal tubule epithelial cell; VEC, vascular endothelial cell; PDMS, polydimethylsiloxane.

Multi-organs-on-chips are sophisticated microfabricated systems that integrate various critical functional units to emulate interactions between multiple organs and achieve human organ-level functions. Given the impact of hepatic metabolism on the nephrotoxicity of certain drugs or chemicals, liver-kidney-on-a-chip models are widely applied in studying drug-induced kidney damage (Figure 4). Choucha-Snouber L *et al.* established a connection between a liver-on-a-chip and a kidney-on-a-chip, allowing culture medium to flow from the liver tissue to the kidney tissue (Choucha-Snouber et al., 2013). In this setup, the liver metabolized ifosfamide into chloroacetaldehyde, which then flowed to the kidney tissue and caused a reduction in MDCK cell numbers (up to 30%). This experiment highlighted that the nephrotoxicity of ifosfamide is mediated by its hepatic metabolites rather than the drug itself. Chang SY *et al.* integrated a kidney-on-a-chip with a liver-on-a-chip to evaluate the impact of hepatic metabolism on aristolochic acid (AA)-induced renal injury (Chang et al., 2017). They found that prehepatic metabolism bioactivated AA-I, resulting in a 5-fold increase in its toxicity to kidney tubular cells. Furthermore, treatment of hepatocytes with the nitroreductase inhibitor dicumarol reduced AA-I-induced nephrotoxicity by 39%. In a subsequent study, the decreased number of GECs caused by verapamil was shown to be milder on the liver-kidney-on-a-chip than on the kidney-on-a-chip (53.7%–69.6% of cellular viability), suggesting that hepatic metabolism can reduce the nephrotoxicity of verapamil (Li et al., 2018). In addition to detecting secondary nephrotoxicity caused by drug metabolism, the liver-kidney-on-a-chip can be employed to study drug-drug interactions. In a simplified liver-kidney-on-a-chip model, biotransformed Aflatoxin B1 (AFB1) reduced kidney cell survival when the fluid flowed from the liver to the kidney (lower than 20% of cellular viability), but its renal toxicity was significantly reduced when the fluid direction was reversed (Theobald et al., 2018). Additionally, a synergistic effect was observed when rifampicin and AFB1 were combined. Similarly, Lin N *et al.* evaluated the hepatic and renal toxicity of cyclosporine A by connecting hepatic spheroids and renal proximal tubules in microfluidic channels. They found that rifampicin reduced the concentration and toxicity of cyclosporine A (Lin et al., 2020). Recently, Nguyen VVT *et al.* established a kidney injury model by combining kidney and liver functional units and investigated the role and distribution of small extracellular vesicles (sEVs) derived from mesenchymal stem cells (Nguyen et al., 2022). The results showed that sEVs could accelerate the recovery of injured renal cells, and renal injury influenced the accumulation of sEVs in the liver. While these studies have made significant progress in understanding drug-induced nephrotoxicity and drug-drug interactions, there is ongoing research to enhance the physiological relevance of liver-kidney-on-a-chip models. This includes the co-culture of multiple cell lines derived from the liver and kidney to better mimic the functional units of these organs. In addition, achieving precise coordination of physicochemical parameters in the two chambers to simulate *in vitro* interactions between the liver and kidney remains a challenging but essential aspect of OoC research.

The development of multi-organs-on-a-chip systems has been pivotal for simulating systemic responses *in vitro*, providing a valuable platform for studying the interactions between multiple organs. Zhang C *et al.* constructed a four-compartment microfluidic platform to culture four different cell types representing various organs: C3A (liver), A549 (lung), HK-2 (kidney), and HPA (fat). Each compartment was isolated to maintain the individual functions of the respective cell types. They observed that TGF-β1 released into the lung compartment enhanced the function of A549 cells without affecting the liver and kidney compartments (Zhang et al., 2009). This multi-channel system recreated four organs and could be used for assessing systemic effects of drugs and screening for food safety, highlighting the importance of inter-organ communication *in vitro*. Maschmeyer I *et al.* developed a multi-organs-on-a-chip incorporating liver, skin, intestine, and kidney compartments. This platform enabled *in vitro* profiling of drug absorption, distribution, metabolism, and excretion as well as repeated dose toxicity testing (Maschmeyer et al., 2015). By connecting these organ compartments, researchers could study how drugs are processed and interact with different organs in a systemic manner. Another study focused on interactions between multiple organs, specifically the jejunum, liver, and kidney, which were infused with a microfluidic medium (Vernetti et al., 2017). This model exhibited the sequential absorption, metabolism, and clearance of three compounds, closely mimicking the *in vivo* process. The data obtained from this platform aligned with organ-specific processing observed *in vivo*, providing insights into multi-organ interactions and drug responses. Miller PG *et al.* developed a highly advanced MPS containing 13 different organs. In this system, various cell types formed barriers to maintain a high survival rate for up to 7 days (Miller and Shuler, 2016). This comprehensive culture system enabled the simulation of drug distribution, metabolism, and action throughout the body. It also facilitated the study of interactions among different cell lines, making it a powerful tool for understanding complex systemic responses. The successful development of this culture system suggests the potential for constructing a complete human body-on-a-chip in the future, which could serve as an alternative to human experiments.

## 4 Chip-based bioengineered kidney

The development of chip-based bioengineered kidneys, particularly implantable artificial kidneys (IAKs), represents a promising advancement in the field of renal replacement therapy (RRT) for patients with end-stage renal disease (ESRD). Traditional treatments such as dialysis and kidney transplantation have limitations, including short life expectancy, reduced quality of life, a shortage of donors, and the risk of immune rejection. Chip-based IAKs offer a potential solution to address these challenges (van Gelder et al., 2018). Microfluidic technology has played a significant role in the development of chip-based IAKs. These systems are designed to be more efficient than traditional dialysis in terms of waste removal and nutrient retention. Microfluidic artificial kidneys have demonstrated greater penetration efficiency *in vitro*, and animal experiments have shown their potential to improve renal function in models of renal failure (Jeffrey et al., 1994; To et al., 2015). In addition, common issues in dialysis, such as thrombus adhesion and protein deposition leading to biofouling, have been addressed in microdialyzer designs (Ota et al., 2016). This is important for maintaining the functionality and longevity of the bioengineered kidney. Over the years, Fissell and his team have worked on developing chip-based IAKs, which consist of kidney cells and a microchip filter. The microchip filter contains a scaffold designed to mimic the structure of kidney cell membranes. This setup enables the separation of metabolic waste from nutrients in the blood, effectively replacing some of the critical functions of natural kidneys (Fissell and Roy, 2009). The research team has made significant progress in examining the biocompatibility of silicon membranes and the differentiation of kidney cells within microelectromechanical devices (Fissell et al., 2006). Chip-based IAKs have several advantages over traditional fiber dialyzers. They are compact, do not require dialysate, and are cost-effective. In addition, these devices use the patient’s blood pressure to drive blood through the filter, reducing the risk of blood clots. They incorporate human-derived biomaterials to minimize the risk of immune rejection and add ECM components to reduce protein adsorption (Dang et al., 2020). Human trials to assess the effectiveness of chip-based IAKs in replacing kidney function are anticipated. Successful outcomes from these trials could potentially offer a path for patients with ESRD to discontinue traditional dialysis.

In summary, chip-based bioengineered kidneys, particularly IAKs, hold promise as a revolutionary approach to treating ESRD. These devices leverage microfluidic technology and innovative designs to provide more efficient and patient-friendly alternatives to traditional dialysis. Ongoing research and human trials are expected to further validate their effectiveness and safety as a RRT option.

## 5 Bioprinted kidney-on-a-chip

**FIGURE 5:**
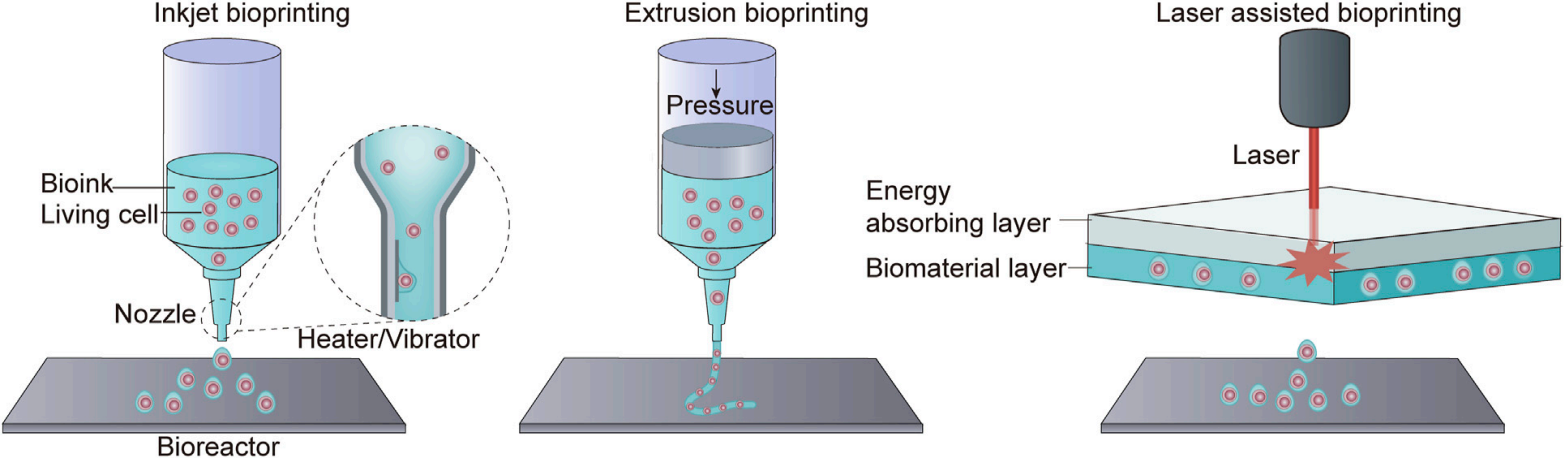
Three bioprinting methods. Inkjet bioprinting uses a heater or vibrator to generate bubbles at the printer’s nozzle, resulting in the spraying of ink droplets into the incubator. Extrusion bioprinting squeezes biological material out of a nozzle through pressure. Laser-assisted bioprinting does not rely on a nozzle. Instead, it employs an energy-absorbing layer to absorb laser energy, allowing for the deposition of biomaterial.

The 3D bioprinting has emerged as an exciting and innovative technology with the potential to revolutionize the field of tissue engineering and regenerative medicine. Bioprinting involves the precise layer-by-layer deposition of biomaterials, including bioink, to create functional 3D tissues and organs. The 3D bioprinting system can read images of tissues or organs and print biological materials into corresponding structures in different methods (Hong et al., 2018). Bioink is a critical component of the bioprinting process. It is typically a water-rich organic solution that contains a mixture of cells and extracellular components (Gungor-Ozkerim et al., 2018). The bioink serves as the “ink” that is used to create the desired tissue or organ structure. There are several bioprinting methods, including inkjet bioprinting, extrusion bioprinting, and laser-assisted bioprinting. Each of these methods has its unique advantages and is suited to different applications. For example, inkjet bioprinting uses thermodynamic or acoustic energy to deposit bioink droplets, while extrusion bioprinting relies on mechanical pressure to extrude bioink. Laser-assisted bioprinting uses laser energy to precisely position bioink (Figure 5) (Hong et al., 2018). The printed original tissue is surgically implanted into the human body after maturity in the bioreactor, or it can be printed directly in the human body to replace damaged or missing tissues or organs (Samandari et al., 2022).

The integration of bioprinting technology and OoC systems has led to significant advancements in tissue engineering and organ modeling. To date, bioprinting has successfully been used to create a wide range of tissues, including skin, bone, heart, and kidney (Michael et al., 2013; Byambaa et al., 2017; Lee et al., 2019; Lawlor et al., 2021). The standard technique for creating kidney tissue is to print a kidney-like structure or tissue-tissue interface and introduce various cell types, including epithelial cells, endothelial cells, and fibroblasts essential for producing the ECM (King et al., 2017; Singh et al., 2020). Bioprinting and OoC complement each other’s strengths. Bioprinting can automatically introduce cells into microfluidic devices, enhancing efficiency and reducing contamination risks (Matsusaki et al., 2013). The precision of bioprinting systems allows for the accurate control of cell placement, improving the fidelity of OoC models (Yamaguchi et al., 2012). PDMS for preparing microfluidic devices absorbs some hydrophobic molecules, thus affecting the accurate measurement of lipid-soluble drug concentration. Bioinks used in bioprinters are typically water-rich and serve as suitable alternatives to PDMS (Li et al., 2021). High-viscosity bioinks used in traditional bioprinting can generate FSS during printing, potentially damaging cell viability. However, recent developments in microfluidic bioprinting have introduced low-viscosity bioinks that allow for the co-extrusion of the bioink and cross-linked solutions. This innovation significantly reduces FSS during the printing process (Davoodi et al., 2020). Therefore, integrating bioprinting technology into microfluidic platforms may be an essential development direction for OoC.

The intersection of bioprinting and OoC has been applied to kidney models. Homan KA *et al.* constructed a programmable proximal tubule-on-a-chip using bioprinting techniques (Homan et al., 2016). The researchers employed fugitive ink deposition on an ECM consisting of fibrinogen and gelatin. Additional ECM was cast around the ink, which was subsequently liquefied and removed to form a hollow tubular channel. This model was then integrated into a perfusable chip, and the channel was seeded with PTECs. Injection of medium facilitated the formation of a 3D proximal tubule structure. Confocal microscopy was used to visualize the tubule structure, while renderings of actin, Na/K-ATPase and tubulin demonstrated the functionality and usability of the model.

Compared to traditional 2D cell cultures, PTECs in this 3D system exhibit significantly improved differentiation phenotype. Researchers have successfully used this model to quantitatively analyze the damaging effects of drugs like cyclosporin A on the epithelial barrier. Cell self-assembly is the spontaneous creation of intricate tissue structures by cells without external guidance, although it occurs with uncertainty regarding both time and space. Tröndle et al. utilized bioprinting techniques to induce controlled self-assembly of PTECs (Tröndle et al., 2021). They bioprinted a continuous luminal pattern with specified orientation, resembling proximal tubules, into a hydrogel ECM. Subsequently, the bioink containing PTECs was embedded in the ECM, allowing it to self-assemble into a cell monolayer lining the lumen following the predetermined pattern. This approach results in tubular structures of predetermined size and location with higher kidney-specific functional gene expression compared to 2D culture. The 3D model was then integrated into microfluidic chips. Opening both ends of the tubule by another self-assembly mechanism and connecting the two ports of the chip through bioprinting to achieve fluidic perfusion along the predefined orientation. The luminal flow rate and FSS measured by polystyrene particle trajectories in the perfusate were consistent with the physiological conditions of rats, further enhancing the imitation of kidney-on-chip models.

The combination of bioprinted and OoC represents a powerful approach to create realistic and functional organ models that can be used for a wide range of applications. With their high throughput capabilities and simulation characteristics, they hold promise for advancing *in vitro* modeling and *in vivo* regeneration studies.

## 6 Current challenges and future perspectives

Although microdevices offer several advantages over conventional *in vitro* models, such as cost-effectiveness, precise control over microenviroments, versatility, and high throughput capabilities, they still face numerous challenges that require urgent attention. In addition to the aforementioned difficulties, such as achieving critical cell differentiation, expressing functional proteins, and replicating complex organ microenvironments *in vitro*, limitations like the absence of an immune system and mesangial cells are also significant. Moreover, various OoCs share common disadvantages. The specialized microengineering fabrication of microfluidic devices can vary among laboratories and even among technicians within the same laboratory (Musah et al., 2018). Thus, there is a pressing need for a more standardized manufacturing model to ensure product consistency. Additionally, cells cultured in OoCs have limited lifespans and are unsuitable for prolonged experiments (Maschmeyer et al., 2015). Researchers, responsible for introducing and separating cells, are inevitable sources of sample contamination. Therefore, there is a growing interest in using computer technology to develop intelligent passive microfluidic systems (Zhang et al., 2024). Furthermore, to investigate how various *in vivo* signals work individually or in combination, it is necessary to adjust the parameters of different chambers, which imposes higher requirements for the systematization of OoCs (Kimura et al., 2018). Consequently, OoCs should not be considered replacements but rather alternatives to traditional animal models until these shortcomings are addressed.

The integration of kidney-on-a-chip with other organs-on-chips has led to the development of a “human-on-a-chip” system. This approach enables the study of interactions among multiple organs and the prediction of pharmacokinetic and pharmacodynamic parameters of drugs in a more holistic manner. The prospect of developing functional, integrated, and vascularized bioengineered kidneys holds promise for replacing traditional therapies like dialysis and kidney transplantation. This approach could also be applied in 3D bioprinting of organs, tissue engineering, and regenerative medicine. OoCs have the potential to identify new biomarkers for drug efficacy, systemic toxicity, and disease response. These biomarkers may find applications in clinical trials, helping to refine drug development processes and reduce the reliance on animal experiments. The ultimate goal is to create a “human-on-a-chip” that integrates multiple organs, accurately replicating human physiology. This approach will enable a comprehensive understanding of how the body responds to drugs and diseases, ultimately replacing animal tests.

## 7 Conclusion

Kidney-on-a-chip models represent a significant advancement in our understanding of kidney biology and offer a promising and innovative approach for studying drug efficacy, toxicity, and disease-related processes in a physiologically relevant context. This review outlines the diverse functional kidney units-on-a-chip and their versatile applications, including drug nephrotoxicity screening, renal development studies, and modeling various kidney diseases to investigate the underlying pathophysiological mechanisms. We also describe the current shortcomings of kidney-on-a-chip models and propose potential treatment strategies. For example, iPSCs facilitate the acquisition of differentiated cells, organoid technology holds promise for promoting organ regeneration and functionalization *in vitro*, and bioprinting technology enhances the production efficiency of chips. As this technology continues to advance, the concept of multi-organs-on-a-chip is emerging, paving the way for the development of human-on-a-chip systems. Specifically, we discuss the synergies between kidney-on-a-chip technology and other emerging biomedical technologies. By integrating with bioprinting and regenerative medicine, kidney-on-a-chip has the potential to revolutionize drug development, disease modeling, and personalized medicine, ultimately benefiting both research and clinical applications. This review aims to provide valuable insights for future researchers and inspire the direction of future work related to kidney-on-a-chip technology.
